# The significance of green entrepreneurial self-efficacy: Mediating and moderating role of green innovation and green knowledge sharing culture

**DOI:** 10.3389/fpsyg.2022.1001867

**Published:** 2022-09-23

**Authors:** Jingyi Guo

**Affiliations:** Law School, Ningbo University, Ningbo, China

**Keywords:** green entrepreneurial self-efficacy, green innovation, environmental performance, economic performance, social performance, knowledge sharing culture

## Abstract

Green entrepreneurial self-efficacy (ESE) refers to individuals’ conviction that they can contribute to solving environmental issues and shows self-assurance in their efforts to protect the environment. The present investigation attempts to determine the role of employees’ green ESE in the green innovation (GI) of SMEs. It is also proposed that GI positively impacts organizational environmental, economic, and social performance. This study also evaluates the mediating role of GI and moderating role of the green knowledge-sharing culture. This study tested the hypothesis using a partial least squares structural equation modeling (PLS-SEM) by applying smart PLS software. A total of 289 employees from SMEs in China were targeted for data collection. The results confirmed that green ESE positively impacts GI. Additionally, the findings verified that GI positively enhances firms’ environmental, economic, and social performance. The results validate the mediating role of GI. The moderating results revealed that green knowledge-sharing culture does not play a moderating role in proposed relationships. This study serves the existing body of literature by providing empirical evidence on the significance of green ESE. The study outcomes highlighted the bridging role of employees’ green ESE for firms’ GI. The results also offer companies a road map for how staff members’ green ESE might help the businesses to improve their performance in terms of the environment, economy, and society.

## Introduction

There are many varying definitions of green entrepreneurship, however, according to the most cited one green entrepreneurship is “the creation of new green enterprise” (Arenal et al., 2020). Given the challenges posed by climate change, there has been immense awareness about green entrepreneurship, however, there are not enough concrete actions to stop or even slow down the process of climate change. The calls for green entrepreneurship in the age of economic development are gaining weight forcing the public and private sectors to establish agencies to pave way for green entrepreneurship. Studies have attempted to find out the characteristics and personal traits to know if they have any role to play in the promotion of green entrepreneurship, however, there has not been any significant breakthrough in this regard that can help carve a prediction model (Prodanova et al., 2021). Nevertheless, a study found that self-efficacy is one of the factors that can have a positive association with an inclination toward green entrepreneurship. Safeguarding the climate and taking the green route is a state of mind that encourages green thinking in entrepreneurs. The purpose of green entrepreneurship is to establish the trend of different entrepreneurship ideas and their positive outcomes (Arenal et al., 2020).

Self-efficacy has been associated with green entrepreneurship and it can be defined as the belief of a person about his/her entrepreneurial capabilities to achieve growth targets while at the same time considering the safety of the environment, moreover, this is also about the belief of the entrepreneur that their economic ventures can be part of a good change. Studies found a correlation between self-efficacy and starting new ventures as self-efficacy promotes positive thoughts that are pivotal to the success of business startups (Rivai et al., 2020; Prodanova et al., 2021). Few constructs were developed by a study such as green marketing, innovation, management, risk-taking, and financial control skills. The study contended that risk-taking and innovation were the constructs that were basic cognitive capabilities that led to entrepreneurial self-efficacy (ESE) (Pushkarskaya et al., 2021). A study concluded that a high level of self-efficacy was positively associated with overcoming business hurdles. Moreover, in a relevant study self-efficacy was found to be a highly important factor in the success of setting up a new business. Self-efficacy is termed as an enabling factor in recognizing the opportunity of an entrepreneurial startup. A study concluded that self-efficacy had positive influences vis-a-vis entrepreneurial intentions (Morsy, 2018).

Degradation of the environment has been recognized as one of the biggest existential threats to the survival of humanity Zhang and Ma (2021). The calls for protecting the environment are blaring loud and green innovation (GI) is trending as it is considered highly significant for environmental protection (Yang et al., 2017). The issue environmental degradation, for instance, has been serious in northern China that includes area such as Beijing, Tianjin, and Hebei. The cluster of small and medium sized enterprises have endangered the environment in this part of China. Therefore, civic agencies have been trying to protect the climate while at the same time ensuring economic advance as well (Ge and Lin, 2021). Importantly, this Chinese environmental dilemma underlines the purpose of this study.

Both at the community and the organizational level, the awareness regarding environmental protection is on the rise, however, there is also this deep realization that along with the environment economic growth is also important. To strike this balance GI initiatives become even more important. Organizations with regard to GI enjoy a competitive advantage (Kawai et al., 2018) since GI is trending, it attracts concerned people and puts one in an advantageous position. According to Schumpeter’s Theory of Innovation GI does help protect the environment that respects the expectations of the customers (Gürlek and Tuna, 2018). Therefore, GI can be defined as the process of achieving economic growth with an aim of protecting the environment from degradation and avoiding resource exploitation (Castellacci and Lie, 2017; Alshebami, 2021).

Innovation is of two types: product/service innovation and innovation of process. The ultimate goal of innovation is to improve the product or service while maintaining a profitable outlook. Therefore, the aim is to welcome innovation that brings cost efficiency and organizational flexibility (El-Kassar and Singh, 2019). Cost efficiency and organizational flexibility entail numerous benefits for an organization such as competitive advantage, cutting down the use of hazardous products, efficient use of resources, innovating eco-friendly practices, introducing eco-friendly products/services, boosting good economic performance, encouraging recycling, mitigating pollution, and conservation of energy (Burki and Dahlstrom, 2017; Huang and Li, 2017; El-Kassar and Singh, 2019). As it is obvious how GI can help organizations and communities improve and gain sustainable advantages (Chu et al., 2019). However, there are quite a few obstacles in the way of an innovation to take place in an organizational setup up. The success of GI depends on knowledge sharing, acceptance of green methods, enforcement of environmental regulations, the commitment of senior management, and incorporation of green corporate culture (Burki and Dahlstrom, 2017; Chu et al., 2019).

Nevertheless, there are numerous challenges in the way implementation of GI innovations such as environmental hazards that ironically come with green technologies, risk of failure in the implementation process, expensive research, and development process, and issues with collection of reliable data, job dissatisfaction among employees, lack of funds, organizations not willing to take risks, not having the appropriate understanding of green initiatives, and lack of governmental support (Abbas and Saðsan, 2019). There is no dearth of studies examining the relation between GI and performance. However, no study has examined the joint effect of GI, ESE, and knowledge sharing. However, it is important to note that some studies do acknowledge a positive relationship between environmental protection practices and knowledge sharing. Knowledge sharing is defined as the flow of knowledge from one unit, individual, or possessor to another. Keeping this definition in view the process of knowledge sharing in an organizational setting deeply influences the performance of all the different segments or units of an organization (Kushnina et al., 2022). Knowledge sharing has always been considered tremendously important for improvement, and betterment, however, the concept has mostly been examined and studied through the lens of individual progress. Nevertheless, now the importance of knowledge sharing has increased manyfold since it has been considered pivotal for advanced managerial applications and organizational theory (Zhang et al., 2018). Now the idea that knowledge sharing is tremendously urgent and important for performance has an empirical foundation (Weidenfeld et al., 2010). Despite the fact that many studies have been conducted in the domain of green entrepreneurship but literature gap still exists to be filled when it comes to examining the roles and relationships of green ESE, GI, firm performance, and knowledge sharing in a single study. The objective of this study is to establish a framework to study the roles and relationships of the above-mentioned variables simultaneously. The study aims to explore answers to the following research questions:

How does green ESE affect GI? How GI influences the sustainable performance of the organization? What role is played by GI between green ESE and the environmental, economic, and social performance of the organization? What role is played by green knowledge-sharing culture between green ESE and GI? After an extensive review of available relevant literature, it was found that no study has been done until now to investigate these research questions. Thus, from the available literature, the current study firstly assumes that green ESE positively impacts GI. Secondly, it is assumed that GI positively impacts environmental performance, economic performance, and social performance of the organization. Thirdly the study assumes the mediating role of GI between green ESE and environmental, economic, and social performance. Fourthly, it assumes that green knowledge-sharing culture moderates the association between green ESE and GI.

The study contributes to the research in the following potential ways: First, the study provides an extensive and comprehensive and systematic overview of the concepts of GI, green ESE, firm performance, and knowledge sharing from the previous research studies. To the best of the author’s knowledge, only a few studies have been done on linking these variables in green entrepreneurship and green management. Secondly, this research contributes through its innovative and unique framework of the study, which proposes, and analyzes the relationships and moderator and mediator roles of the variables of this study. Thirdly, the study contributes by adding green knowledge sharing culture into the integrated analytical framework that studies the relationship between green ESE, GI, and organizational performance. The framework of this study explains the theoretical perspective in an innovative way. The study also has theoretical and practical implications. Theoretically, it extends the literature on resource-based view (RBV) theory while practically it offers guidelines for the stakeholders of green entrepreneurship.

The remaining part of the current study is outlined as follows: section “Literature review and hypotheses development” of this research study presented the overview of available literature on GI, knowledge sharing, and green self-efficacy, and green performance. Applied methods and analysis have been portrayed in section “Research methodology.” Section “Results” presented statistical interpretation and empirical results. In the end, the discussion, conclusion, and theoretical and practical implications have been summarized.

## Literature review and hypotheses development

### Theoretical support and background

There are multiple methods to view and gauge the advantage and performance of a firm, however, according to the theory of RBV, it depends on how a firm utilized its strategic resources (Barney, 1991; Jayasinghe et al., 2022). The competitive advantage rest on the nature of the strategic resources. For instance, if the available resources are not imitable and rival firms do not have any means to carve up alternative resources of the same value and function then the competitive advantage will be long-lasting yielding superior performance in the shape of achieving targets (Bhandari et al., 2022). This research argues that self-efficacy, green knowledge and employees are the strategic resources as we apply theory of RBV to ESE. The study contends that these resources fulfill the criteria of theory of RBV by enabling higher performance and competitive advantage.

### Green entrepreneurial self-efficacy and green innovation

Self-efficacy is referred to as one’s belief in his/her abilities to achieve the expected outcomes using skills and resources. A study found that self-efficacy was a crucial prerequisite for achieving pro-social behavior. Subsequently, the same was found true for entrepreneurial behavior as well (Pong et al., 2005). Now as far as the matter of measurement of self-efficacy is concerned it depends on the problem as well as the field involved. Therefore, self-efficacy with regard to green entrepreneurship refers to one’s belief about his/her abilities and confidence to solve the issue of environmental degradation (Chu et al., 2021).

Though green entrepreneurship is emerging, however, it is starkly different from ordinary entrepreneurship because of two particular aspects. Firstly, its dependence on the green market as well green consumer base is what separates it from ordinary entrepreneurship (Lotfi et al., 2018). The second aspect is that of policy orientation on which green entrepreneurship depends heavily. As green entrepreneurship tends to have greater environmental and social responsibilities, and simultaneously has to handle the issue of a longer payback period as well, therefore, encouraging policy regimes is highly important for green entrepreneurship encouragement (Chu et al., 2021). The requirements in sense of responsibility are high in the case of green entrepreneurship as compared to ordinary entrepreneurship since there is not only the issue of economic requirements that need to be considered but an additional requirement of social and environmental responsibilities must also be taken care of Aghelie (2017).

Value choices in green entrepreneurship matter the most and these value choices are determined by an individual’s perspective about their attachment and relationship with not only the environment but also with society as well (Yang et al., 2017). Ecological guidelines steer one’s relationship with the environment. There are two important aspects of the current ecological value research. Firstly, there are structural dimensions of ecological values and there are different opinions regarding the nature of these dimensions such as believed that these values were monodimensional and contrarily contend that these values were multidimensional (Dunlap et al., 2000; Amburgey and Thoman, 2012). The second aspect is about the factors that affect the ecological values. And there is an array of different factors ranging from demography to literacy rate, from social background to gender, etc. that shape an individual’s ecological values (Mac Lennan et al., 2021).

Apart from ecological values, the sense of social responsibility is another dimension that is highly crucial in green entrepreneurship. A study aptly put it that green entrepreneurship is a behavior that has multiple targets and this behavior operates in an uncertain environment (Riillo, 2017). Multiple-target implies that environmental responsibilities and entrepreneurial responsibilities are fulfilled simultaneously. Therefore, the social mission of the people at the helm is given the central position while formulating a conceptual model of green entrepreneurship (Afsar et al., 2018).

Those entrepreneurs who have environmental ambitions embedded in their sense of social responsibility tend to vigorously pursue their business goals and not give up in the wake of challenges. The intensity and vigor of the green entrepreneurship intentions strongly depend on the green ESE and entrepreneurship abilities of the intention holders (Alvarez-Risco et al., 2021). The adaption and improvement of green products and the green process are at the core of GI. It is also included the technologies that aim at conservation and protection of the environment by consuming less energy, spreading less pollution and adopting green design regimes (Yang et al., 2017). Since across the board acceptability toward GI, the idea has attracted increased attention as it ensures a win-win situation that guarantees better, sustainable environmental protection as well as advancement in innovation, and progress.

**H1:** Green entrepreneurial self-efficacy positively impacts green innovation.

### Green innovation and its impact on organizational performance

The vision of a firm for sustainability crucially rests upon the role of the entrepreneurs who lead firms. The impact of entrepreneurial orientation and ESE are the two factors that have been heavily focused on by different studies that examined the factors behind sustainable performance (Marshall et al., 2005). Recently, the focus of the research has shifted to the entrepreneurial attributes that are found in a firm along with the urge for innovation (Wagner, 2011). As the fate of competitive advantage more and more rests on green entrepreneurship, modern research tends to tilt toward shifting its focus on the link between entrepreneurship attributes of a firm and its environment-friendly business models. One such study examined the impact of readiness of the firms with regards to corporate entrepreneurship on its financial performance. The study found that the impact of corporate entrepreneurship significantly impacted financial as well as environmental performance. Another study revealed that green entrepreneurship was followed by positive growth in the financial development of the firms (Wagner, 2011).

A firm’s bid to go beyond societal expectations in meeting its environmental responsibilities shapes its environmental performance indicators It is not only about compliance with governmental regulations, however, but it also encompasses environmental effects that the operations of a firm cast (Dubey et al., 2015; Oliva et al., 2019). The agenda of a firm regarding environmental management highlights the extent of GI in a particular firm. GI is at the helm while it provides the stimulation for environmental performance (Adegbile et al., 2017).

On the other hand, green processes, and innovation not only mitigate the negative impact on the environment but also enhances both the financial and social performances of a firm as cost-effective measure are adopted as a result of GI (Weng et al., 2015). Therefore, studies have argued that GI better not be termed as a reactive measure of the firms in the wake of outside regulatory pressure rather it should be considered as the voluntary innovative behavior of the firm founded on the basis of achieving competitive advantage over competitors (Kratzer et al., 2017). Therefore, considering RVB, the study predicts that green entrepreneurship is considered a highly valued resource by firms that have serious potential to help a firm achieve their environmental targets and good repute (Dragomir, 2020).

The competitive advantage of a firm depends on its valuable organizational resources (Barney, 1991). From the RVB theory’s perspective incorporating environmental aspects to the goals of an entrepreneurial venture strategic resources of a firm get a consolidated result in competitive advantage (Lannelongue et al., 2017). Moreover, a deeper environmental management orientation places a firm in a better position to develop unique resources as compared to the firms that engage themselves superficially to the environmental cause. Studies revealed that comprehensive and holistic environmental protection policies enable firms to achieve better economic prospects (Swalehe et al., 2020; Chu et al., 2021).

A deep sense of social responsibility lies at the bottom of green entrepreneurship intentions. The extent of a sense of social responsibility determines the level of commitment of an entrepreneur toward his/her green business goals. As there could be multiple challenges down the corporate road and this sense of social responsibility enables the entrepreneurs to stick to their green entrepreneurship intentions and not make a compromise in protecting the environment. Firms with environmental management outlook tend to have a close relationship with other stakeholders such as the government, environmental organizations and the target community. This closer relationship reduces the cost of risk management for the firms (Gürlek and Tuna, 2018). Moreover, well-designed and innovative products cost low but have a higher value. This particular aspect enables firms to utilize their resources optimally and become even more competitive (Kawai et al., 2018). Furthermore, socially responsible firms enjoy the trust of banks, financial institutions and investors which ultimately gives much-needed financial breathing space to the firms to expand both vertically and horizontally (Aghelie, 2017). Better environmental management of the firms can also prove to be beneficial for the social image of the firm. Employees can have enhanced identification toward such a firm which translates into better prospects for the firm to both attract and retain talent and solve the problem of high labor costs (Weng et al., 2015). Studies show that green products and processes positively affect the competitive advantage of entrepreneurial ventures (Chen et al., 2021).

**H2:** Green innovation positively impacts environmental performance.

**H3:** Green innovation positively impacts economic performance.

**H4:** Green innovation positively impacts social performance.

### Green innovation as a mediator between green entrepreneurial self-efficacy and environmental performance

Organizations having implemented green innovativeness tend to have a higher success rate. The overall performance of such organizations is also better than their competitors because of their readiness to adapt to the needs of their customers. This readiness adds value to the organization resultantly (Chen et al., 2015a). Moreover, the firms which have a genuine commitment to environmental protection seem to do well both environmentally and economically. Furthermore, green initiatives of a firm allow it to develop its products in a better way that positively affects the green developmental culture of the business (Weng et al., 2015). Moreover, green ESE is also found to provide impetus to the cause of environmental awareness, promote the green performance of the firm and increase green creativity. Though there has been evidence that green self-efficacy influences GI, however, the aspect of handling the regulatory pressure while having green environmental intentions still needs to be empirically supported by further studies (Jia et al., 2018). It is further suggested by multiple studies that to continue for a firm to execute its green operation green recruitment standards need to be adopted to ensure that fresh employees share the same environmental commitment as the firm itself and there is no discrepancy as far as environmental goals of a team of people are concerned (Dragomir, 2020).

The importance of having a clear vision regarding the current and future trajectory of the firm is paramount for green entrepreneurs since the nature of the market is mostly dynamic and a clear vision help navigate amidst challenges (Dragomir, 2020). The onus of responsibility for having a concrete and clearer vision rests on the leaders of entrepreneurial ventures, additionally, studies recommend that the concrete vision, and trajectory course should be explicitly elaborated to the entire team so that they can know the importance of the vision and be excited about achieving green targets (Chen et al., 2015a). The same study suggests that such a shared vision enables higher motivation, commitment, and better performance. Studies have shown that leaders having a favorable belief system at the core of their intellect regarding green entrepreneurship positively influences the overall operations of the firm from a better performance at all levels to talent and resource management (Jia et al., 2018). This is a compounding process during which GI leads to a further increase in green efficiency of the firm enhancing environmental commitment further (Afsar et al., 2018). Therefore, green ESE is shown to have a key role in carving out a clear vision, recruiting employees in accordance with the vision of the leader, striving for GI, and adapting to the green policies and practices to get as close to the stated goals as possible (Jia et al., 2018).

**H5**: Green innovation mediated the relationship between green entrepreneurial self-efficacy and environmental performance.

**H6**: Green innovation mediated the relationship between green entrepreneurial self-efficacy and economic performance.

**H7**: Green innovation mediated the relationship between green entrepreneurial self-efficacy and social performance.

### Green knowledge sharing culture as a moderator between green entrepreneurial self-efficacy and green innovation

The process of innovation heavily depends on the knowledge pool of employees at both collective and individual levels. Furthermore, the utilization of the available knowledge is also key to the process of innovation. Moreover, employees’ skills and experience in creating value in a system are also one of the main key aspects of the process of innovation. Keeping these aspects in view, the extent of knowledge sharing in a firm determines how innovative that firm can be with its ideas, policies and products/services (de Sousa et al., 2020). As the process of innovation heavily depends on knowledge, therefore, the willingness, and capabilities of the employees to better share and manage the flow of knowledge determine how well-positioned an organization is to achieve its organizational goals in terms of innovation. The process of innovation required employees to utilize both explicit and implicit knowledge at hand. Therefore, the culture of knowledge sharing in a firm determines how innovative and efficient that particular firm is (Peng, 2013).

The culture of environmental management in an organization is formed by the basic characteristics of the firm (Fauji and Utami, 2013). The vision of sustainability of a firm determines how the firm uses technology and manages its knowledge. The motivation for ecological improvement emanates from the culture of knowledge sharing with the subsequent counterparts. This leads to enhanced cooperation among all stakeholders and efficient attainment of the strategic vision of green entrepreneurship. Furthermore, knowledge sharing is considered the fundamental aspect of any firm’s success and prosperity (Rodgers et al., 2017)

A study by examined the relationship between innovation of the firm, financial and operational performance, and knowledge sharing (Wang and Wang, 2012). The study concluded that tactic knowledge sharing was positively linked with both the financial as well as the operational performance of an organization. However, as far as explicit knowledge sharing was concerned it was found to have a positive link only with the financial performance of the firm. The same study contended that the speed of innovation had a positive impact on both financial and operational performance, whereas, the results of the quality of innovation were different from the speed of the innovation as it only impacted the financial performance of the firm positively and did not show any considerable positive link with the operational performance of the firm.

**FIGURE 1:**
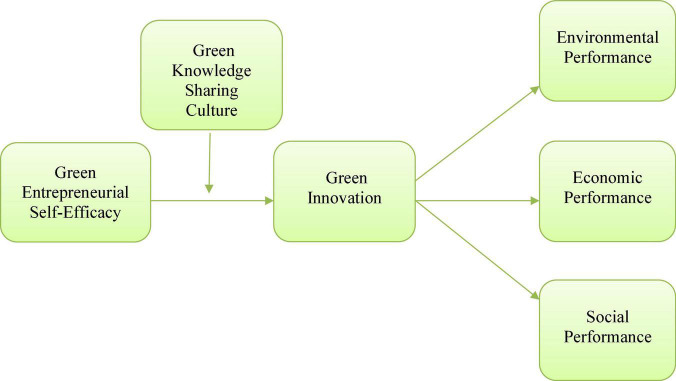
Research model.

Another study examined the impact of knowledge sharing in the realm of joint ventures. The study concluded that knowledge sharing was a significantly key factor in the performance of entrepreneurial ventures. Furthermore, the same study also highlighted how the process of knowledge sharing eliminated drawbacks that obstruct the flow of knowledge particularly in joint ventures. A rather recent study examined the association among different factors such as knowledge sharing innovation, environmental management, and product/service quality (Hamdoun et al., 2018). This study also concluded that the practice of environmental management was positively associated with knowledge sharing. Furthermore, it also came to for that there was a positive link between knowledge sharing and GI. The research framework of this study is given in Figure 1.

**H8:** Green Knowledge sharing culture moderates the positive association between green entrepreneurial self-efficacy and green innovation.

## Research methodology

### Measurement

#### Independent variable

Green entrepreneurial self-efficacy is an independent construct of the present study, measured through a three items scale. The scale was particularly designed by Hockerts (2017) and then adapted and used in the context of green ESE by Wang et al. (2021). The sample item included, “I believe that if I do it with my heart, I can contribute to the environment (see Appendix A).” Its Cronbach alpha value is 0.899.

#### Mediator variable

The green innovation construct is taken in this study as a mediator and measured with seven items scale adapted from Chen et al. (2006) and validated by Singh et al. (2020). Four items were related to green product innovation, and three were related to green process innovation. The sample item included for green product innovation was “My company uses materials that produce the least pollution,” for green process, innovation was, “The manufacturing processes of my company effectively reduce hazardous substance or waste.” The Cronbach alpha value is 0.897.

#### Dependent variable

The sustainable performance was a dependent construct of the present study, which was measured on fifteen items scale including three performances such as environmental, economic, and social. Each performance has five items. This scale is taken from Khan and Quaddus (2015). This scale was also validated by Iqbal et al. (2018, 2020). The sample item included for environmental performance was “My organization uses utilities (e.g., energy and water) in an environmentally friendly manner,” for economic performance was “Economic Performance of my organization is at an acceptable level in terms of sales growth,” and for social performance was “My organization enhances your social recognition in the society.” The Cronbach alpha value of environmental, economic, and social performance was 0.922, 0.898, and 0.883, respectively.

#### Moderator variable

The present study uses the green knowledge-sharing culture as a moderator construct. This construct was measured based on a five items scale adapted from (Wong, 2013; Rubel et al., 2021). The sample item included was “I enjoy sharing environment-friendly knowledge with my colleagues.” The Cronbach alpha value was 0.860.

### Data collection

China is facing a severe threat of environmental problems. The China Northern district is called as Bohai Bay economic circle. This district includes Beijing, Tianjing, and Hebei Province. Hence, due to the large scale of small and medium enterprises (SMEs) in this district, China is facing high pollution in this area (Zeng et al., 2011). Undoubtedly, SMEs have significantly played a role in social and economic development, but unfortunately, they also created severe environmental problems. Hence, the present study targeted the manufacturing sector of SMEs for data collection for empirical analyses of green self-efficacy and its impact on sustainable performance. The present study randomly selected 50 SMEs and contacted them through email and phone. The reminder email was also sent to get permission from all SMEs after 1 week. But most of them did not give a positive response. Hence, the present study approached those SMEs who positively responded and agreed to corporate in our research work data collection. Upon the permission of seventeen SMEs managers, the author visited personally and distributed 450 questionnaires among employees. In addition, the surveys were translated into two languages: English and Chinese. A native Chinese speaker performed the translation. A panel of senior researchers approved the final questionnaires before they were used in the data-gathering process. A letter explaining the purpose of the research was delivered to the participants along with the detail of their data privacy and usage. The participants were also confident about the right or wrong answers. This step boosted their confidence and helped in gathering natural responses. Out of 450 responses, the present study received 328 questionnaires from the participants. After scrutinizing the verified missing and incomplete filling of the questionnaires, the present study finalized the 289 complete and valid responses for conducting empirical analyses. The response rate of the present study is 64.22% confirming that a good response such as, according to the prior study, 35.5% response was considered satisfactory for empirical analyses (Baruch and Holtom, 2008).

## Results

### Statistical model

Hair et al. (2019) point out that structural equation modeling (SEM) is one of the most suitable statistical methods for data analysis. Covariance-based (CB-SEM) and variance-based partial least squares structural equation modeling (PLS-SEM) are the two main methods of SEM (Hair et al., 2014). PLS-SEM was used in this investigation to analyze the data statistically. The main justification for this choice is PLS-SEM’s applicability to confirmatory and exploratory investigations (Hair et al., 2016). With no explicit requirements for data normality, PLS-SEM is a suitable method for complicated and multi-order models. PLS-SEM is also appropriate for analyzing small data sets (Bashir et al., 2021). Therefore, the PLS-SEM approach is taken into account in the current study while utilizing Smart PLS software to analyze empirical data.

### Model measurement

The model’s reliability is validated by using the values of Cronbach’s alpha, roh-A, composite reliability (CR), and average variance extract (AVE) (Hair et al., 2014). According to the threshold level set by Hair et al. (2011), the values for Cronbach’s alpha, roh-A, CR, and AVE should be greater or equal to 0.7. Table 1 depicts that all values meet the necessary standards. The AVE values are used to determine the convergent validity of constructs. The acceptable criterion for AVE is that the values must fulfill the set standard, which is 0.5 or above. The AVE values are bigger than 0.5, as seen in Table 1. Therefore, the convergence validity of the variables is thus established.

**TABLE 1:** Measurement reliabilities.

Construct	Composite reliability	roh-A	Cronbach’s alpha	AVE
Economic performance	0.924	0.899	0.898	0.710
Environmental performance	0.941	0.923	0.922	0.762
Green entrepreneurial self-efficacy	0.937	0.901	0.899	0.831
Green innovation	0.919	0.898	0.897	0.618
Green knowledge sharing culture	0.899	0.867	0.860	0.641
Social performance	0.915	0.888	0.883	0.683

**FIGURE 2:**
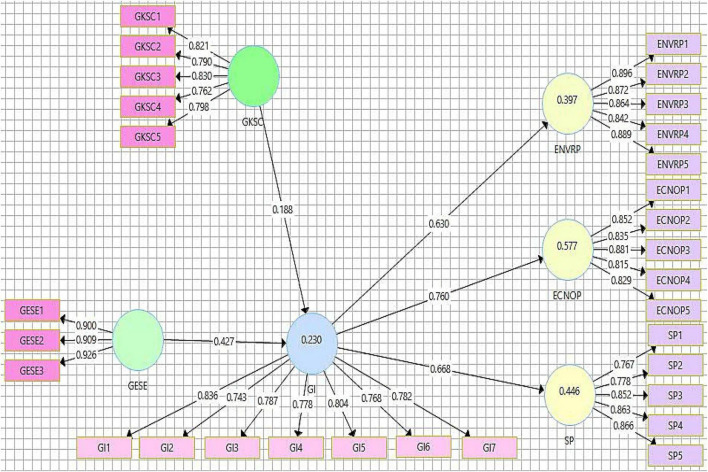
Path model.

Each item’s outer loading value is considered to weigh the constructs’ reliability (Hair et al., 2013). Values must be greater than or equal to 0.7 to meet the threshold for factor loading. According to Table 2, all outer loading values are in accordance with the threshold level (Figure 2). The VIF results are analyzed to confirm the model’s collinearity. The model is deemed to be free of collinearity issues if the VIF values are smaller than 0.5 (Hair et al., 2019; Bashir et al., 2021). According to information depicted in Table 2, the maximum VIF value is (GESE-3) 3.369, meaning that all values are below 0.5. Thus, it is demonstrated that the model has no collinearity problems.

**TABLE 2:** Outer model loadings.

Construct	Name	No. of items	Items deleted	Items	Outer loadings	VIF
Dependent	Economic performance	5	None	ECNOP1	0.852	2.491
				ECNOP2	0.835	2.252
				ECNOP3	0.881	2.869
				ECNOP4	0.815	2.139
				ECNOP5	0.829	2.226
Dependent	Environmental performance	5	None	ENVRP1	0.896	3.252
				ENVRP2	0.872	3.030
				ENVRP3	0.864	2.700
				ENVRP4	0.842	2.521
				ENVRP5	0.889	3.087
Independent	Green entrepreneurial self-efficacy	3	None	GESE1	0.900	2.376
				GESE2	0.909	3.017
				GESE3	0.926	3.369
Mediator	Green innovation	7	None	GI1	0.836	2.619
				GI2	0.743	2.019
				GI3	0.787	2.085
				GI4	0.778	2.144
				GI5	0.804	2.173
				GI6	0.768	1.983
				GI7	0.782	2.092
Moderator	Green knowledge sharing culture	5	None	GKSC1	0.821	1.892
				GKSC2	0.790	1.982
				GKSC3	0.830	2.207
				GKSC4	0.762	1.930
				GKSC5	0.798	2.040
Dependent	Social performance	5	None	SP1	0.767	2.182
				SP2	0.778	2.238
				SP3	0.852	2.694
				SP4	0.863	2.994
				SP5	0.866	2.834

The validity of the constructs was assessed in this study using the heterotrait-monotrait (HTMT) ratios and the Fornell-Larcker criterion. By finding the square roots of the AVE values for the model constructs, the Fornell-Larcker criterion is established (Hair et al., 2016). The data satisfy the Fornell-Larcker criterion if the first value on each column’s upper side is greater than the values on its bottom side. Table 3 reveals that all Fornell-Larcker criteria values meet the predetermined standards. Hair et al. (2019) note that in order to confirm the specifications of HTMT ratios, all values of HTMT must be smaller than 0.85. Table 4’s findings reveal that the constructs’ HTMT values are smaller than 0.85, which suggests that the model used for the current analysis has demonstrated discriminant validity (Hair et al., 2017).

**TABLE 3:** Fornell-Larcker criterion.

Constructs	ECNOP	ENVRP	GESE	GI	GKSC	SP
ECNOP	0.843					
ENVRP	0.650	0.873				
GESE	0.443	0.439	0.912			
GI	0.760	0.630	0.442	0.786		
GKSC	0.167	0.066	0.079	0.221	0.801	
SP	0.623	0.486	0.395	0.668	0.174	0.826

Green entrepreneurial self-efficacy, green innovation, environmental performance, economic performance, social performance, green knowledge sharing culture.

**TABLE 4:** Heterotrait-Monotrait ratio (HTMT).

Constructs	ECNOP	ENVRP	GESE	GI	GKSC	SP
ECNOP						
ENVRP	**0.716**					
GESE	0.494	**0.477**				
GI	0.842	0.686	**0.484**			
GKSC	0.186	0.082	0.098	**0.250**		
SP	0.702	0.542	0.442	0.750	**0.201**	

Green entrepreneurial self-efficacy, green innovation, environmental performance, economic performance, social performance, green knowledge sharing culture. Bold values represent the relationship between variables.

According to the evaluation of the *R* square values, latent variable values more than or near 0.5 indicate the model’s moderate strength, while values closer to 0.25 indicate its weak strength (Hair et al., 2019). The Figure 2 describes the *R*^2^ value for GI (0.230) shows weak model strength, while the *R*^2^ values for ENVRP (0.397), ECNOP (0.577), and SP (0.446) show moderate model strengths. Cross-validated redundancy (*Q*^2^) values for the model are deemed significant if they are more than zero (Hair et al., 2014). This study’s latent variables all had *Q*^2^ values greater than zero, which is another encouraging sign of a robust model.

### Structural model evaluation

The bootstrapping technique with 5,000 samples is utilized to conduct the empirical analysis for this study. In the present study, the focus was given to the statistical “*t*” and “*p*” values for accepting and rejecting hypotheses. Table 5 displays the results for direct connections. The findings of the first hypothesis (*t* = 7.011, *p* = 0.000) certified that green ESE positively impacts GI. The path value informed that green ESE caused 0.312 variations in GI. The H1 is accepted on the basis of statistics described in Table 5. The results of H2 (*t* = 8.046, *p* = 0.000) verified that GI positively impacts environmental performance, which authenticates that H2 is accepted. The beta value of H2 is 0.630. The outcomes of the third hypothesis show that (*t* = 12.987, *p* = 0.000) GI enhances economic performance. The H3 is accepted with a path value of 0.760. According to results Table 5 (*t* = 8.150, *p* = 0.000), H4 is accepted, which confirms that GI positively impacts social performance. The path value of the fourth hypothesis is 0.668.

**TABLE 5:** Direct relationship.

Relationship	Path value	Mean	Standard deviation	*T* dtatistics	*P*-values	Hypotheses outcomes
GESE → GI	0.312	0.310	0.044	7.011	0.000	H1, accepted
GI → ENVRP	0.630	0.620	0.078	8.046	0.000	H2, accepted
GI → ECNOP	0.760	0.753	0.058	12.987	0.000	H3, accepted
GI → SP	0.668	0.655	0.082	8.150	0.000	H4, accepted

Green entrepreneurial self-efficacy, green innovation, environmental performance, economic performance, social performance, green knowledge sharing culture.

### Mediation and moderation results

The results of mediation analysis are described in Table 6. The results show that (*t* = 4.897, *p* = 0.000) GI mediates the association between green ESE and environmental performance. The H5 is accepted with the path value of 0.197. The H6 of this study is also accepted (*t* = 5.698, *p* = 0.000) with a path value of 0.237, which is the verification of the mediating role of GI in the association between ESE and economic performance. The results presented in Table 6 further revealed that GI mediates the correlation between green ESE and social performance (*t* = 5.024, *p* = 0.000). The H7 is accepted with the path value of 0.208.

**TABLE 6:** Indirect relationship.

Relationship	Path value	Mean	Standard deviation	*T* statistics	*P*-values	Hypotheses outcomes
GESE → GI → ENVRP	0.197	0.193	0.040	4.897	0.000	H5, accepted
GESE → GI → ECNOP	0.237	0.234	0.042	5.698	0.000	H6, accepted
GESE → GI → SP	0.208	0.204	0.041	5.024	0.000	H7, accepted

Green entrepreneurial self-efficacy, green innovation, environmental performance, economic performance, social performance, green knowledge sharing culture.

**FIGURE 3:**
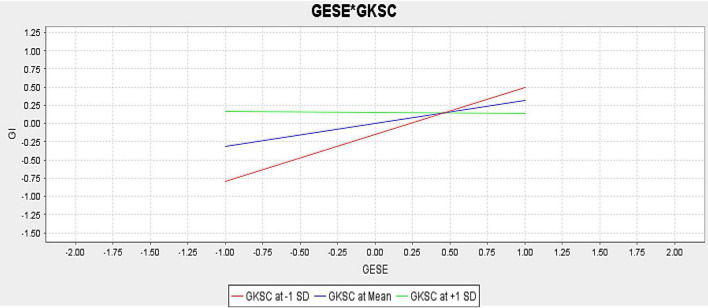
Moderation slope of green self-efficacy*green knowledge sharing culture.

The moderation results are presented in Table 7. For empirical investigation of moderating role of green knowledge-sharing culture, this study proposed that green knowledge-sharing culture positively moderates the association between green ESE and GI. However, the findings of H8 *t* statistics (5.787), and *p* statistics (0.000) with a negative path value (−0.327) revealed that green knowledge-sharing culture does not moderate the association between green ESE and GI. Therefore, H8 is rejected. The moderation slope is given in Figure 3.

**TABLE 7:** Moderation relationship.

Relationship	Path value	Mean	Standard deviation	*T* statistics	*P*-values	Hypotheses outcomes
GESE*GKSC → GI	−0.327	−0.315	0.057	5.787	0.000	H8, rejected

Green entrepreneurial self-efficacy, green innovation, green knowledge sharing culture.

## Discussion

Entrepreneurial self-efficacy plays a favorable role in driving organizational sustainability and performance (Ahmed, 2020). Hmieleski and Baron (2008) also illuminated the importance of ESE and said that ESE could play an essential role in boosting firms’ working efficiency and effectiveness. Wang et al. (2021) highlighted the significance of green entrepreneurship self-efficacy and stated that it is the belief that one can solve environmental problems and demonstrates self-assurance in one’s efforts to save the environment. Employees with high ESE tend to set tough objectives, work hard to attain those goals despite challenging circumstances, and bounce back quickly from demanding circumstances (Hmieleski and Baron, 2008). Based on resource-based review theory, this study aims to determine the importance of employees’ green ESE for firms’ environmental, economic, and social performance. The current investigation also evaluates the mediating role of GI and the moderating role of green knowledge-sharing culture. This study proposed eight hypotheses to test the relationships empirically. According to the first hypothesis, green ESE positively impacts GI. The second, third, and fourth hypotheses stated that GI positively relates to environmental, economic, and social performance. The fifth, sixth, and seventh hypotheses depicted that GI mediates the connection between green ESE and environmental, economic, and social performance. The eighth hypothesis of this study proposed that green knowledge-sharing culture positively moderates the association between green ESE and GI.

According to findings, H1 is accepted, demonstrating that green ESE positively impacts GI. These results align with earlier research (Chen et al., 2015b; Akhtar et al., 2021; Farooq et al., 2022). These studies highlighted the importance of employees’ green ESE for firms’ GI. Moreover, sustainable business practices and social progress may be achieved *via* effective GI. Farooq et al. (2022) stated that GI is essential for businesses to adapt to green trends and gain a competitive edge. Environmental management may aid businesses in the green era not only in overcoming obstacles but in instigating green creativity (Akhtar et al., 2021). Additionally, employees with green ESE could assist the firms in gaining desirable consequences for firms’ green performance. Businesses with a first mover in GI may gain competitive benefits while reducing their manufacturing waste and industrial pollution.

The finding of this study further verified that GI has a positive role in boosting firms’ environmental performance. Hence, it is confirmed that the second hypothesis of the current investigation is accepted. These revealing have consistency with prior studies (Chiou et al., 2011; Singh et al., 2020; Rehman et al., 2021). These investigations point out that the reduction of environmental harm, and protection against resource exploitation are two areas where GI substantially influences environmental performance. Rehman et al. (2021) acknowledged that an organization’s environmental strategy and proactive tactics to create eco-friendly technology might boost its environmental effectiveness.

The results of the third and fourth hypotheses depicted that GI positively impacts firms’ economic, and social performance, respectively. Therefore, H3 and H4 of this research are also accepted. These findings have consistency with prior studies (Antonioli et al., 2016; Iqbal et al., 2018; Zhang et al., 2018). These studies pointed out the importance of GI and stated that a company’s economic and social performance is favorably correlated with GI. Businesses may utilize it to boost productivity and offset growing raw material costs. New technologies and procedures are fundamentally altering conventional business practices into GI, which greatly lowers their harmful effects on the environment. GI may also result in the development of new goods and procedures that aid in the sanitization and restoration of the environment (Rehman et al., 2021).

According to mediation relationships findings, the fifth, sixth, and seventh hypotheses are accepted, which means that GI has a mediation role in the relationship between green ESE and environmental, economic, and social performance, respectively. The findings have consistency with prior studies (Chen et al., 2015b; Zailani et al., 2015; Ahmed, 2020; Asadi et al., 2020). These studies argued that employees’ green ESE assists the firms in enhancing their GI, which in turn leads to an increase in firms’ environmental, economic, and social performance. The idea of using GI as a key organizational resource could help with a variety of social and environmental problems connected to sustainable business practices (Khanra et al., 2022). Moreover, utilizing GI as a corporate resource might give your company long-term competitive benefits due to lower production costs. Watts et al. (2021) point out that the achievement of green and vigorous industrial growth depends on GI. Additionally, GI works as a link between environmental law and the innovative modernization of industrial companies. Takalo and Tooranloo (2021) argued that the success of businesses and communities in terms of the environment, and economy, as well as their ability to respond to environmental rules, have all been linked to GI.

The moderating results depicted that green knowledge-sharing culture does not moderate the association between green ESE and GI. These findings show that the H8 of this study is not accepted. The reason behind this rejection may be the weak knowledge-sharing culture of the firms, as Islam and Asad (2021) noticed that the weak knowledge-sharing culture of the firm slowed down the innovation activities of SMEs. Song et al. (2020) further acknowledged that green knowledge-sharing culture boosts firms’ GI and sustainability. Rubel et al. (2021) acknowledged that green knowledge sharing culture has a well-established impact on a number of performance outcomes, including enhanced client connections, high-quality services, and innovative performance. Moreover, employees that participate in a culture of knowledge sharing pass on their expertise to coworkers, which leads to the creation of more collaborative knowledge inside the company. According to Wong (2013), the performance of individuals and teams is favorably correlated with the organization’s information-sharing culture.

## Implications

The theoretical implications of this study introduce an advancement in the theoretical framework itself. The study advances and validates RBV (Barney, 1991) with the assistance of an empirical explanation of why entrepreneurial ventures take the route of GI recognizing the bigger environmental scenario and still successfully carrying out financial operations. The study based on its empirical results suggests that green ESE be considered as the strategic resource that can help firms design, implement green policies, and practices. Furthermore, we suggest, while applying RVB, in the light of the results of this study that green ESE, as well as green knowledge, should be treated as valuable resources so that they may not be imitated by competitors to gain strategic advantage. It is further suggested by this study that green practices have to be innovated to the extent that they can attract green employees who later can be trained and retained by ensuring that all the units in the firm remain strictly aware of the green goals and entrepreneurial significance of those goals. This study also reveals the mediating and moderating roles of knowledge sharing and GI.

Our study suggests that to examine the environmental performance of an entrepreneurial venture, the nature of the green process, and the quality of GI are the key factors. Therefore, it is suggested that both these factors are to be implemented not under pressure from the stakeholders, but rather in a proactive manner to aim at the strategic goal of maintaining competitive advantage while at the same time protecting the environment as well. Innovation is suggested as an intangible asset that can bring the aforementioned competitive advantage and enhance the performance of the company (Gürlek and Tuna, 2018). Therefore, GI also works on the same principles with all the potential to create prospects for a firm. Furthermore, it is suggested that innovation be systematically improved to cut the production cost and make the products/services efficient and cost-effective.

## Limitations

Although the present study serves the literature in multiple ways by providing a unique body of knowledge; however, there are still some limitations. These limitations may provide opportunities and directions to future researchers. First, this study assumes the employees’ green ESE as an antecedent of GI and firms’ environmental, economic, and social performance. In the future, researchers may use other antecedents like green entrepreneurial motivation and green entrepreneurial intentions. Second, this study determines the role of green ESE on organizational environmental, economic, and social performance; future studies may research the impact of employees’ green ESE on firms’ energy efficiency. Third, the current study assesses the moderating role of green knowledge-sharing culture in the correlation between green ESE and GI. Future studies may consider entrepreneurial orientation as a moderator to validate the study results. Fourth, the current study’s findings were only inferred from 289 employees’ replies, which is a small sample size. This model can eventually be validated using large comparative data. Finally, the structured questionnaire approach was utilized in this study to gather data; however, other data collection methods, such as interview techniques, may be used in the future.

## Conclusion

The employees’ characteristics of ESE are seen to be important among the managerial characteristics of the company. Self-efficacy of employees as green entrepreneurs helps companies to develop creative corporate environments, strengthen relationships with stakeholders, support important company goals, and overcome environmental challenges. This study aims to investigate the influence of green ESE on GI using the RBV theory. This study further proposed that GI positively affects firms’ environmental, economic, and social performance. The present investigation also evaluates the mediating role of GI and moderating role of the green knowledge-sharing culture. The results illustrated that green ESE positively correlates with GI. The results demonstrated a strong correlation between GI and an organization’s performance in terms of the environment, economy, and society. The findings show that GI is a mediator between green ESE and environmental, economic, and social performance. The results also showed that the relationship between GI and green ESE does not moderate by green knowledge-sharing culture. This investigation revealed the importance of employees’ green ESE for firms’ GI. The results also assist firms in boosting their employees’ green ESE to improve their environmental, economic, and social performance.

## Data availability statement

The original contributions presented in this study are included in the article/supplementary material, further inquiries can be directed to the corresponding author.

## Ethics statement

The studies involving human participants were reviewed and approved by the Ningbo University, China. The patients/participants provided their written informed consent to participate in this study. The study was conducted in accordance with the Declaration of Helsinki.

## Author contributions

JG: conceptualization, data collection, writing the draft, and agree the submitted version of manuscript.

## Appendix

Appendix A | Measuring items.

### Green entrepreneurial self-efficacy

1- I believe that if I do it with my heart, I can contribute to the environment.

2- I can find a way to help solve environmental problems.

3- Solving environmental problems is a contribution that each of us can make.

### Green innovation

#### Green product innovation

1- My company uses materials that produce least pollution.

2- My company uses materials that consumes less energy and resources.

3- My company uses materials that to design environment friendly product.

4- My company uses materials that are easy to recycle, reuse, and decompose.

#### Green process innovation

5- The manufacturing processes of my company effectively reduces hazardous substance or waste.

6- The manufacturing processes of my company effectively reduces consumption of coal, oil, electricity or water.

7- The manufacturing processes of my company effectively reduces use of raw materials.

### Green knowledge sharing culture

1- I enjoy sharing my knowledge with colleagues.

2- I enjoy helping colleagues by sharing my knowledge.

3- It feels good to help my colleagues by sharing my knowledge.

4- Sharing my knowledge with colleagues is pleasurable.

5- I believe knowledge sharing can benefit all parties involved.

### Sustainable performance

#### Economic sustainability

1- We see our firm is providing employment to us and others.

Our firm’s economic performance is at an acceptable level in terms of …

2- sales growth.

3- income stability.

4- return on investment.

5- profitability.

#### Social sustainability

Our firm…

6- ensures basic needs for our family.

7- enhances our social recognition in society.

8- improves our empowerment in society.

9- provides freedom and control over the course of our own lifestyle.

10- is concerned about child labor use.

#### Environmental sustainability

Our firm…

11- uses utilities (e.g., energy and water) in an environmental friendly manner.

12- produces few wastes and emissions.

13- is concerned about waste management.

14- uses small space to set up and operate business.

15- is concerned about hygienic factors.
